# Enrolment of families with overweight children into a program aimed at reducing childhood obesity with and without a weight criterion: a natural experiment

**DOI:** 10.1186/s12889-019-6894-y

**Published:** 2019-06-14

**Authors:** Emma Esdaile, Emely Hernandez, Carly Jane Moores, Helen Anna Vidgen

**Affiliations:** 1 School of Exercise & Nutrition Sciences, Faculty of Health, Queensland University of Technology, Level 4, A Wing, O Block, Kelvin Grove, Brisbane, Queensland Qld 4059 Australia; 2 Nutrition and Dietetics, College of Nursing and Health Sciences, Flinders University, GPO Box 2100, Adelaide, South Australia 5001 Australia

**Keywords:** Childhood obesity, Primary school, Healthy lifestyle program, Engagement, Eligibility

## Abstract

**Background:**

Difficulties engaging families with overweight children to enrol into programs aimed at reducing childhood obesity have been well documented. During the implementation of the Parenting, Eating and Activity for Child Health Program (PEACH™) over a large geographical area (Queensland (QLD), Australia), a natural experiment developed. This experiment provided an opportunity to observe if there was a difference in enrolment for families with overweight children with a weight criterion (referred to as the period with a Targeted Eligibility Criterion (TEC)) compared to when a weight criterion was removed (the period referred to as Universal Eligibility Criterion (UEC)). We also examined the eligibility criterion’s relationship with attendance, parental concern about their child’s weight, estimation of overweight and obesity from parent-reported data.

**Methods:**

A secondary analysis of baseline data from 926 overweight/obese children from 817 families enrolled in PEACH™ QLD was performed. Analyses were adjusted to control for the presence of clustered data. Bivariate statistics were performed using Pearson chi-square test with the second-order Rao-Scott correction, and Mann–Whitney U-test for non-parametric continuous variables. Generalized Estimating Equations (GEE) explored the association between weight status-based eligibility criteria and enrolment of overweight children. GEE were adjusted for sex, age and socioeconomic index and stratified for weight category.

**Results:**

Compared to obese children, overweight children were almost twice as likely to be enrolled when the program did not have weight status-based eligibility criteria (during UEC period) (OR = 1.90 (CI 95% 1.35–2.68, *p* <  0.001)). Parents of overweight children enrolled during the UEC period were more likely to regard their child’s weight as less of a concern than during the TEC period (UEC 67% vs. TEC 45%, *p* = 0.036). Children whose parent-reported data underestimated their weight category were more likely to be enrolled while the program did not have weight-related eligibility criteria OR = 2.27 (CI 1.38–3.70, *p* <  0.01). Program session attendance did not appear to be impacted by the changes in eligibility criteria.

**Conclusions:**

The omission of weight criteria for healthy lifestyle programs is a consideration for health professionals and decision-makers alike when encouraging the enrolment of children who are overweight into healthy lifestyle programs.

**Trial registration:**

ACTRN12617000315314. Retrospectively registered 28 February 2017.

**Electronic supplementary material:**

The online version of this article (10.1186/s12889-019-6894-y) contains supplementary material, which is available to authorized users.

## Background

Elevated obesity prevalence rates are an international phenomenon, and one in four Australian children aged 5–17 years are overweight or obese [1]. Body Mass Index (BMI) categories of excess weight reflect the different levels of risk of chronic illness experienced by overweight and obese individuals. Obesity contributes to multiple co-morbidities in childhood and adulthood, as well as with all-cause mortality when compared to normal or overweight status [2–5], and increases lifetime risk of developing chronic disease [6]. As such, returning children to their healthy weight range is likely to have the greatest health benefits relative to the risks associated with lifelong excess weight. While childhood weight management programs likely have benefits irrespective of the child’s weight status at enrolment, those who are overweight, rather than obese, are more likely to shift their weight status category into the healthy range [7], giving them the healthiest foundation for adulthood [8, 9]. This reinforces the importance of early identification and intervention.

The Parenting, Eating and Activity for Child Health (PEACH™) Program is an evidence-based [7, 10] healthy lifestyle program that was scaled up and delivered statewide in Queensland (a geographically large state of Australia) to parents of primary school-aged children who were above their healthy weight range. In Queensland, the prevalence of overweight in children aged 5–17 years is 19%, while the obesity prevalence is 7% [11]. Despite the higher proportion of overweight compared to obese children in the general population, the children who enrolled into PEACH™ Queensland (PEACH™ QLD) and were above a healthy weight were predominantly obese (79%) rather than overweight (21%) [10]. Other studies show difficulties in recruiting families with overweight children into weight management programs in Australia; when parents enrol, their children tend to be disproportionately obese, rather than overweight [10, 12]. Despite these difficulties PEACH™ QLD was interested in increasing enrolments among children who were overweight, in order to better reflect the target population.

From a public health perspective weight loss among obese and overweight children is significant. However, the probability of returning to a healthy weight is higher [7] while avoiding the development of co-morbidities is lower [13] for children who are overweight (compared to obese) and so maximising their enrolments into healthy lifestyle programs is a key prevention strategy. Early qualitative research undertaken among parents who enrolled into PEACH™ QLD identified that parents sought out a range of other methods to act on their child’s weight before they considered enrolling into a weight management program [14], suggesting there are factors which delay enrolment as children continue to gain excess weight. Continuous quality improvement during the scaling up of PEACH™ QLD (described in detail elsewhere [15]) provided the opportunity for a retrospective natural experiment to explore whether parents of overweight children were more likely to enrol into the program when the weight status eligibility criterion was removed.

Our research aim was to investigate if a weight criterion acts as a barrier to enrolment into healthy lifestyle programs aimed at reducing childhood obesity. Three key themes emerge from the literature as the primary barriers for parents of overweight children not enrolling into a weight management program. These barriers include perceived stigma [16–18], inability of parents to recognise their child is above their healthy weight [19, 20] and parents not considering weight to be an immediate health issue [20, 21]. While stigma was not measured, our evaluation framework collected data that relates to the other identified barriers. These were the extent of agreement between parent-reported and facilitator-measured anthropometric data and the extent to which parents were concerned about the seriousness of obesity for their child.

We hypothesised that the removal of a weight criterion would lower barriers to entry and proportionately more parents with overweight children would enrol, rather than delaying seeking external support for their child. To explore this, we compared the proportion of overweight and obese children enrolled into the program before and after the removal of weight status-based eligibility criterion to be above a healthy weight. We compared factors that related to identified barriers to enrolment, where data were available. We also compared enrolment and attendance before and after the weight-status eligibility criterion to observe if there was a difference in these characteristics in order to inform recruitment for future programs.

## Methods

### PEACH™ Queensland

The Queensland Government contracted Queensland University of Technology to deliver the PEACH™ Program using a license from its creators [10], this project is referred to as PEACH™ QLD [15]. We have previously described implementation learnings [15], evaluation [22] and program outcomes [7] of PEACH™ QLD elsewhere. Briefly, PEACH™ consists of ten 90-min group-based face-to-face sessions delivered by a trained facilitator over a six-month period. The parent group sessions focus on parenting skills training to improve the healthy lifestyle behaviours (diet, physical activity and sedentary behaviour including screen time) of children [23]. Consistent with clinical practice guidelines [24], PEACH™ QLD focused on healthy lifestyle messages using whole-of-population messages from the Australian Dietary Guidelines [25] and Australia’s Physical Activity & Sedentary Behaviour Guidelines for Children [26] rather than focus on weight reduction explicitly. As such, its content was suitable for all children irrespective of weight status. Facilitators then developed parents’ skills in recognising the obesogenic environment and developing strategies to guide their family in adopting healthy behaviours. Children participate in concurrent facilitated sessions which include non-competitive physical activities and a brief healthy lifestyle activity designed to complement the content of the parent sessions.

The primary objectives of PEACH™ QLD were to enrol 1100 children and to deliver the program as broadly across the state of Queensland as possible [15]. Eligibility to enrol into the program originally required children to reside in Queensland, be 5–11 years of age, and be above a healthy weight range for their age and gender. The PEACH™ QLD project was delivered in five waves across more than 3 years and changes to eligibility are summarised in Table 1.

**Table 1:** Summary of PEACH™ QLD timeline with targeted versus universal eligibility criteria

	Targeted Eligibility Criterion (TEC)	Universal Eligibility Criterion (UEC)
Program Wave	Waves 1–4	Wave 5
Date range	October 2013–April 2016	February – September 2016
Age criterion	5–11 years	Primary school aged^a^
Weight criterion	Above healthy weight only	All weight categories were accepted
Residence	Families must reside in Queensland	

^a^In Queensland, children are able to commence primary school from 4 years, depending on when their date of birth falls in the calendar year. The age criteria (5–11 years) meant that some primary school aged children were not eligible to enrol. In Wave 5 the extension to ‘primary school age’ was made to streamline recruitment strategies [16]

Waves one and two (October 2013 to April 2015) were primarily focused on the piloting of the program across different healthcare settings, with a secondary focus on geographical reach. Waves three and four (February 2015 to April 2016) heavily focused on geographical reach, and smaller towns reported finding it more difficult to recruit participants [15]. Between 2014 and late 2015 several meetings were held with program facilitators and informal discussions were held with participants as part of the quality improvement cycle to identify strategies for improving program delivery. Also, health professionals in towns with close-knit communities identified that the removal of a weight criterion could reduce the potential stigma of attending a weight management program and may encourage more families to enrol and attend sessions. There were also concerns that the program was not reaching those most likely to successfully transition to their healthy weight range. After consideration of this feedback and in consultation with the funder, the eligibility criteria for child weight status was removed for the final roll out phase, Wave 5 (February to September 2016). In this paper, we refer to Waves one to four as the TEC phase and Wave five as Universal Eligibility Criterion (UEC) phase in relation to these differences in eligibility relating to child weight status, as in Table 1.

Messages within the PEACH™ Program focused on healthy lifestyle and not specifically on weight loss, so program content was not changed and the removal of the weight-based eligibility criterion meant that the program acted as both a primary and secondary prevention intervention in the UEC groups. Marketing and advertising materials were also changed: original marketing materials during the TEC made reference to ‘healthy weight’ while later UEC marketing messages focused on ‘healthy lifestyle’ with no reference to weight.

## Measures

### Demographics

Demographic data, including child age, gender, height and weight, postcode and source of referral, were collected at the first point of contact with parents in order to assess eligibility for enrolment. Parent Indigeneity was also determined at enrolment, in line with best practice [27]. Social disadvantage, and accessibility and remoteness were determined using the postcode-based scores: Index of Relative Socio-Economic Disadvantage (IRSD) [28] and the Accessibility/Remoteness Index of Australia (ARIA2011+) [29], respectively. The IRSD is a component of the Socioeconomic Indexes for Areas (SEIFA) [28], where the highest IRSD quintile indicates a relative lack of disadvantage in general, and the lowest quintile indicates a greater level of disadvantage in general [28]. ARIA2011+ is a measure of geographical remoteness or accessibility for every location in Australia, and it can be classified as five categories, that range from highly accessible to very remote. Both measures were used as categorical and continuous variables in this study.

### Recruitment and weight status

Referrals into the program came in two ways: either parents self-referred or they were referred by the health sector (health professionals or directly from a hospital wait list, see [15] for a more detailed description of these referral methods). Health professional-reported or parent-reported child height and weight were collected for the duration of the program. During the phase of the program with a weight status eligibility criterion (referred to as the Target Eligibility Criterion (TEC) phase) these reported child measures were used to assess the eligibility for enrolment into the program, set at or above the 85th percentile for child age and gender, calculated using US-CDC BMI growth charts [30]. Self-referral was initiated through a comprehensive marketing strategy whose messages were modified as a result of change in eligibility criteria (details reported elsewhere [15]).

Children also had their height, weight, and waist circumference measured by trained child facilitators when they attended their first session. The method of weight measurement used for the PEACH™ QLD program has been described previously [7]. Children with biologically implausible values for anthropometric data, defined by the US-CDC [30], were excluded from all analyses as described in a previous paper [7]. These 17 cases (1.8%) were all generated by parent-reported child measures. Facilitator-measured data were available for 700 children who attended the program (75.6% of enrolled children above their healthy weight). In the present study, parent- or health professional-reported anthropometric data obtained at enrolment were used for children without facilitator-measured height and weight (*n* = 226).

The agreement between parent-reported data at enrolment, and facilitator-measured data at program sessions was explored for the subsample with available data (*n* = 551). These measures were compared to determine the relationship of parental reporting biases (i.e., if they were aware of their child’s current weight and height) with enrolment into the program. For this analysis, the parent-reported anthropometric data was classified as over-reporting, under-reporting or agreement. These classifications refer to parent reporting of child height and weight, not the reporting of child weight status. Over-reporting was defined as parent-reported height and weight resulting in a weight status category higher than that derived from facilitator-measured data (i.e., parent-reported data was calculated to result in an obese/ overweight category, when the facilitator-measured data was calculated to result in overweight/ normal weight category). In contrast, under-reporting was defined as parent-reported height and weight resulting in a weight status category lower than that derived from facilitator-measured data (i.e., parent-reported data was calculated to result in underweight/ normal weight/ overweight, when facilitator-measured data was calculated to result in normal weight/ overweight/ obese result). Finally, agreement meant that the information parents provided at enrolment, when calculated, was in agreement with the measured data.

### Program attendance

Trained facilitators who delivered PEACH™ recorded parent attendance at each session and classified children as ‘attended’ if their parents attended at least one session and ‘never attended’, if parents did not attend any sessions. Families were able to enrol until the third session. For those who attended, the total number of sessions attended was calculated as a range from 1 to 10.

### Parental perceptions

Parent’s perceptions regarding the severity of their child’s weight status were captured at baseline. Parents were asked: “Do you think that your child’s weight is a serious health condition?” Parents responded to each item using a 5-point Likert scale (end points: not serious, very serious). These responses were re-coded into 3 categories: not serious (answers 1 and 2), serious (answer 3), very serious (answers 4 and 5).

### Sample and statistical analysis

Children above their healthy weight at enrolment were selected in order to directly compare differences between participants in PEACH™ QLD with UEC and TEC. These children were classified as overweight or obese (including children classified as obese/morbidly obese according to IOTF extended) [31].

The program allowed for multiple children from the same family to be enrolled into the program. This led to a mixture of children enrolled alone (without siblings), and children enrolled with one or more siblings. In these cases, observations from children from the same family were not independent from one another for some variables. Consequently, our methods have been adapted to account for potential cluster effect of the presence of siblings in the dataset. Specifically, Pearson chi-square test with the second-order Rao-Scott correction and Generalized Estimating Equations (GEE) were used instead of conventional Pearson chi-square and logistic regression, respectively.

The proportion of categorical variables were compared by enrolment phase (TEC vs UEC) using Pearson chi-square test with the second-order Rao-Scott correction. Total number of sessions attended, a non-parametric continuous variable, was compared by the enrolment of TEC and UEC phase using the Mann–Whitney U-test. Chi-square analyses conducted to compare the proportion of children enrolled in the TEC and UEC stages of the program in terms of their parents’ perceptions of the seriousness of their weight, included adjusted residuals which indicate the magnitude of the difference between observed and expected counts. Analyses evaluating attendance and parental concern for their child’s weight were performed in the whole sample as well as stratified for weight category, in order to detect possible confounding effects.

Family-level data from the subset of 99 families that enrolled more than one child was evaluated with the aim of determining if overweight children were recruited along with obese siblings, as opposed to being independently recruited. A conventional Pearson chi-square analysis was used to compare the proportions of families that enrolled exclusively overweight siblings, exclusively obese siblings or a combination of both weight categories during the UEC and TEC phases.

To account for clustering of siblings at the family level, GEE logistic regression analyses with robust standard errors, were used to assess the relationship between UEC enrolment and weight category, as well as the agreement of weight category calculated from parent-reported data with categories derived from measures taken by facilitators. GEE has been shown to be an appropriate analysis strategy for datasets with intra-cluster dependence and small, unbalanced clusters [32–34]. GEE models were computed using an exchangeable correlation matrix structure with a binomial probability distribution and a logit link function. Both children and families were entered as subject variables in the model.

The GEE logistic regression analyses were conducted in several steps. First, we estimated the unadjusted odds ratios for each relationship. Potential confounding variables for this relationship were selected a priori based on the literature and their association with the predictor and the outcome, and then sequentially included in the model. Evaluated confounders were: age, sex, and SEIFA score.

Data were entered and analysed in SPSS version 21 (SPSS Inc., Chicago, Ill., USA) where test results with *p* ≤ 0.05 were considered to be statistically significant.

## Results

A total of 926 children above a healthy weight were enrolled in PEACH™ QLD from 817 families.

Characteristics of children are presented in Table 2. Overall, most children were obese, from highly accessible geographical areas and from areas with a relative lack of disadvantage. Over two-thirds (76%) of children were self-referred into the program by their parents or carers. In order to assess the effect of modifying the program eligibility criteria to include all weight categories, we compared children enrolled when the program had weight category eligibility criteria (TEC, waves 1–4) with children enrolled during the time when the program did not have weight category eligibility criteria (UEC, wave 5), see Table 1. When compared to children enrolled at the time of TEC, children enrolled at the time of UEC were more likely to be overweight (30% vs. 20%, *p* = 0.001), male (57% vs. 43%, *p* <  0.001), living in highly accessible areas (ARIA) (82% vs. 69%, *p* = 0.012), referred to the program by a health professional (21% vs. 14%, *p* <  0.001) and have parents identify as Aboriginal or Torres Strait Islander (7% vs. 5%, *p* < 0.001), see Table 2. With the exception of accessibility index (ARIA), these differences stayed significant when data from children with facilitator-measured anthropometry were analysed (Table 2).

**Table 2:** General characteristics of overweight and obese children enrolled in PEACH™ QLD, and their families

	Anthropometric data collection							
	All participants with available anthropometric data^a^ (*n* = 926)				Participants with anthropometric data measured by facilitator (*n* = 700)			
	All	Targeted Eligibility Criterion	Universal Eligibility Criterion	*p*	All	Targeted Eligibility Criterion	Universal Eligibility Criterion	*p*
Child characteristics	*n* (%)	*n* (%)	*n* (%)		*n* (%)	*n* (%)	*n* (%)	
Sex								
Boys	435 (47.0)	297 (43.5)	138 (56.8)	< 0.001	319 (45.6)	229 (43.3)	90 (52.6)	0.037
Girls	491 (53.0)	386 (56.5)	105 (43.2)		381 (54.4)	300 (56.7)	81 (47.4)	
Age at baseline								
< 8 years old	302 (32.6)	212 (31.0)	90 (37.0)	0.185	234 (33.4)	174 (32.9)	60 (35.1)	0.845
≥ 8 -< 10 years old	297 (32.1)	222(32.5)	75 (30.9)		236 (33.7)	179 (33.8)	57 (33.3)	
≥ 10 years old	327 (35.3)	249 (36.5)	78 (32.1)		230 (32.9)	176 (33.3)	54 (31.6)	
Weight category (IOTF 2012)^b^								
Overweight	207 (22.4)	135 (19.8)	72 (29.6)	0.001	553 (79.0)	98 (18.5)	49 (28.7)	0.005
Obese and morbidly obese	719 (77.6)	548 (80.2)	171 (70.4)		147 (21.0)	431 (81.5)	122 (71.3)	
ARIA								
Highly accessible	675 (72.9)	475 (69.5)	200 (82.3)	0.012	505 (72.1)	369 (69.8)	136 (79.5)	0.204
Accessible	91 (9.8)	73 (10.7)	18 (7.4)		69 (9.9)	56 (10.6)	13 (7.6)	
Moderately accessible	120 (13.0)	100 (14.6)	20 (8.2)		93 (13.3)	75 (14.2)	18 (10.5)	
Remote/Very remote	40 (4.3)	35 (5.1)	5 (2.1)		33 (4.7)	29 (5.5)	4 (2.3)	
SEIFA quintile (IRSD)								
Most disadvantaged	113 (12.2)	77 (11.3)	36 (14.8)	0.637	82 (11.7)	57 (10.8)	25 (14.6)	0.820
IRSD quintile 2	162 (17.5)	118 (17.3)	44 (18.1)		121 (17.3)	90 (17.0)	31 (18.1)	
IRSD quintile 3	173 (18.7)	125 (18.3)	48 (19.8)		137 (19.6)	105 (19.8)	32 (18.7)	
IRSD quintile 4	332 (35.9)	250 (36.6)	82 (33.7)		244 (34.9)	188 (35.5)	56 (32.7)	
Least disadvantaged	146 (15.8)	113 (16.5)	33 (13.6)		116 (16.6)	89 (16.8)	27 (15.8)	
Referral source								
Self-referral	710 (76.7)	523 (76.5)	187 (77.0)	0.001	559 (79.9)	424 (80.2)	135 (78.9)	0.008
Wait list	70 (7.6)	66 (9.7)	4 (1.6)		52 (7.4)	48 (9.1)	4 (2.3)	
Health professional	146 (15.8)	94 (13.8)	52 (21.4)		89 (12.7)	57 (10.8)	32 (18.7)	
Parental Indigeneity status								
Aboriginal and/or Torres Strait Islander	50 (5.4)	33 (4.8)	17 (7.0)	< 0.001	37 (5.3)	29 (5.5)	8 (4.7)	0.028
Not Aboriginal and/or Torres Strait Islander	724 (78.2)	510 (74.7)	214 (88.1)		632 (90.3)	470 (88.8)	162 (94.7)	
Not stated/Not recorded	152 (16.4)	140 (20.5)	12 (4.9)		31 (4.4)	30 (5.7)	1 (0.6)	

^a^Parent-reported child and weight were used in the absence of anthropometric data measured by facilitators

*p* values obtained using second-order Rao-Scott adjusted chi-square statistic

^b^Overweight, obese and morbidly obese, as classified by IOTF (2012) [23]. Obese and morbidly obese have been combined in one category

To explore whether the enrolment of overweight siblings of obese children was a contributing factor to the greater proportion of overweight children during UEC, analysis of a subset of families (*n* = 99 families) with more than one child enrolled and available weight data, was performed (data not shown). During UEC a greater proportion of families with only overweight children were enrolled (UEC 15% vs. TEC 2%, *p* = 0.001). On the other hand, a greater percentage of families with a combination of obese and overweight children enrolled during UEC (UEC 57% vs. TEC 37%, *p* = 0.001), at the expense of a smaller proportion of families that only enrolled obese siblings (UEC 27% vs. TEC 61%, *p* = 0.001).

A total of 551 children had both parent-reported anthropometric data at enrolment and facilitator-measured height and weight from sessions. IOTF weight categories derived from parent-reported data were compared to those obtained from facilitator-measured anthropometric measurements to estimate the agreement of these measurements. A median period of 42 days (IQR = 19–86) elapsed between parent-reported and facilitator-measured anthropometric data. For the majority of children (73.3%, *n* = 404), parent-reported data was in agreement with facilitator-measured data. Weight category calculated from parent-reported data was underestimated in 16.9% (*n* = 93) and overestimated in 9.8% (*n* = 54) of children in the subsample with these available data.

Table 3 shows the results of GEE logistic regression analyses performed to determine if being enrolled during the UEC stage of the program was associated with weight status. After adjusting for sex, age, and a socioeconomic index (SEIFA IRSD), children who were overweight were nearly twice as likely to enrol during the UEC stage of the program than during the TEC, when compared to children who were obese (OR = 1.90 (CI 95% 1.35–2.68, *p* < 0.001)). Exclusion of children without facilitator-measured anthropometric data from the regression model did not change these results substantially (Table 4).

**Table 3:** Odds Ratios for the association between Universal Eligibility Criterion and child weight category, sequentially adjusted for potential confounders (*n* = 926)

Predictor of enrolment	Universal Eligibility Criterion^c^		
Weight category	Unadjusted OR (95% CI)	Model 1^a^ OR (95% CI)	Model 2^b^ OR (95% CI)
Overweight	1.71 (1.22–2.39)^**^	1.88 (1.34–2.65)^***^	1.90 (1.35–2.68)^***^
Obese	Reference	Reference	Reference

**p-value < 0.01, ***p-value < 0.001

All values obtained using generalized estimating equation (GEE) methods

^a^Model 1, adjusted for child’s sex and age at baseline

^b^Model 2, Model 1 + SEIFA score

^c^Reference = Targeted Eligibility Criterion

**Table 4:** Odds Ratios for the association between Universal Eligibility Criterion and two predictors: child weight category (facilitator-measured data only), and agreement of parent-reported child weight category, sequentially adjusted for potential confounders

Predictor of enrolment	Universal Eligibility Criterion^c^		
	Unadjusted OR (95% CI)	Model 1^a^ OR (95% CI)	Model 2^b^ OR (95% CI)
Weight category, measured (*n* = 700)			
Overweight^c^	1.76 (1.19–2.63)^**^	1.88 (1.25–2.81)^**^	1.90 (1.27–2.85)^**^
Obese	Reference	Reference	Reference
Agreement of weight status derived from parent-reported data (*n* = 551)			
Under-reported	2.26 (1.39–3.68)^**^	2.26 (1.39–3.68)^**^	2.27 (1.38–3.70)^**^
Over-reported	1.25 (0.64–2.46)	1.25 (0.64–2.45)	1.25 (0.64–2.46)
Agreement	Reference	Reference	Reference

**p-value < 0.01

All values obtained using generalized estimating equation (GEE) methods

^a^Model 1, adjusted for child’s sex and age at baseline

^b^Model 2, Model 1 + SEIFA score

^c^Reference = Targeted Eligibility Criterion

Further, children whose parent-reported anthropometric data yielded a lower weight category than the one obtained from facilitator-measured data (i.e., under-reported their child’s weight category), were two times more likely to enrol during the UEC stage, when compared to children whose weight category derived from parent-reported data showed agreement with facilitator-measured weight category (OR = 2.27 (CI 1.38–3.70, *p* < 0.01), Table 4).

### Attendance

**Fig. 1:**
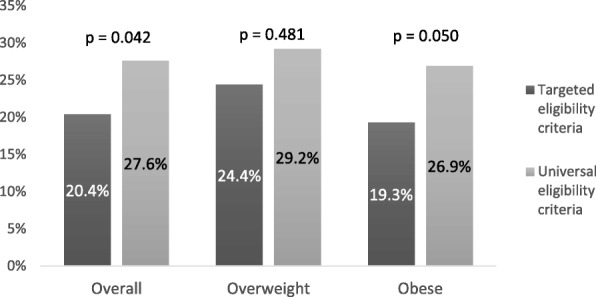
Percentage of children who never attended by eligibility criteria. *p* values obtained using second-order Rao-Scott adjusted chi-square statistic. Overall sample is n = 926; overweight n = 207 and obese n = 719

A significantly higher percentage of children above their healthy weight enrolled at the time of UEC did not attend any sessions when compared to those enrolled in groups with TEC (27.6% vs. 20.4%, *p* = 0.042). After stratifying for weight status category, this association appeared to be statistically significant for obese children (26.9% vs. 19.3%, *p* = 0.050), but not for overweight children (29.2% vs. 24.4%, *p* = 0.481) (Fig. 1).

For children that attended one or more sessions (*n* = 720), a Mann-Whitney U-test indicated that the total number of sessions attended was marginally, but significantly greater for children enrolled in groups with UEC (Mdn = 7, IQR = 4.25–9, Mean Rank = 387) than for children enrolled in groups with TEC (Mdn = 7, IQR = 3–9, Mean Rank = 352), U = 43,178.5, *p* = 0.049 two-tailed.

### Parental perceptions about child weight

A significant relationship was observed between the type of eligibility criteria at the time of a child’s enrolment and the parental concern about their child’s weight (Table 5). During the UEC stage, parents were significantly more likely to consider their child’s weight less of a concern (‘not serious’: UEC 35% vs. TEC 25%, *p* = 0.044). After stratifying for weight category, this association was only significant for parents of overweight children (UEC 67% vs. TEC 45%, *p* = 0.036). Repeating these analyses including only children with facilitator-measured anthropometric data led to comparable results for overweight children (Additional file 1: Table S1). Conversely, when the weight status was calculated based on parent-reported measures only, there were no significant differences among any of the groups (Additional file 1: Table S2).

**Table 5:** Cross-tabulation of parental perception of the seriousness of their child’s weight by eligibility criteria at enrolment, and weight status

		Do you think that your child’s weight is a serious health concern?								
		All children (*n* = 709)			Overweight children (*n* = 150)			Obese children (*n* = 559)		
Phase		Not serious	Serious	Very serious	Not serious	Serious	Very serious	Not serious	Serious	Very serious
Targeted Eligibility Criterion	*n* (%)^a^	135 (25.5)	199 (37.6)	195 (36.9)	43 (44.8)	39 (40.6)	14 (14.6)	92 (21.2)	160 (37.0)	181 (41.8)
	AR^b^	−2.3	1.7	0.6	−2.6	2.5	0.6	0.04	0.6	−0.5
Universal Eligibility Criterion	*n* (%)^a^	63 (35.0)	55 (30.6)	62 (34.4)	36 (66.7)	12 (22.2)	6 (11.1)	27 (21.4)	43 (34.1)	56 (44.4)
	AR^b^	2.3	−1.7	−0.6	2.6	−2.5	−0.6	−0.04	−0.6	0.5
		χ2 = 6.409, df = 2*,* *p* = 0.044			χ2 = 6.895, df = 2, *p* = 0.036			χ2 = 0.377, df = 2, *p* = 0.836		

*p* values obtained using second-order Rao-Scott adjusted chi-square statistic

^a^Row percentages

^b^AR adjusted residuals, ARs over 2 and below − 2 indicate statistically significant differences

## Discussion

The aim of this study was to identify if proportionately more overweight children were enrolled into a parent-led lifestyle program following the removal of weight status-based eligibility criterion to be above a healthy weight. Here we have shown that it was more likely for an overweight child to be enrolled into PEACH™ QLD when UEC were applied. Children whose parent-reported data were classified as under-reported, were more likely to enrol during the UEC stage. Our results also indicate that there was a significantly higher rate of children who were obese that never attended any sessions during the UEC phase of PEACH™ QLD compared to the TEC. Overall for all children above a healthy weight who attended at least one session, attendance was slightly (and significantly) better for the groups with UEC, indicating that having universal weight eligibility criteria did not dissuade ongoing attendance of families with children above their healthy weight. Our sample was representative of the Queensland population in terms of age, gender, socioeconomic status, and Aboriginal and Torres Strait Islander status.

Three barriers to enrolment in child obesity programs are reported in the literature; perceived stigma of participating in such a program [16–18], inability of parents to recognise their child is about their healthy weight [19, 20] and parents not considering weight to be an immediate health issue [20, 21]. Although not measured in the present study, the literature supports the likelihood that perceptions of stigma are a barrier to program enrolment [16, 17]. Parent concern about stigma can manifest in different ways, they may be concerned that their child will feel the consequences of being stigmatised for their weight [20, 21], or they may feel isolated or blamed for their child’s obesity status [18]. Stigma may be a barrier to enrolment because parents are reluctant to identify their child as being overweight [20]. The stigma associated with obesity may be enough for parents to avoid programs aimed at weight management altogether [20]. Parents may have experienced weight stigma themselves in childhood, either from health professionals or their own family, and want to spare their own children from potential lasting emotional consequences [35]. They may also be concerned about effecting their child’s self-esteem [20] or developing unhealthy eating behaviours [35]. The reduction of stigma is an important potential implication of eliminating the weight criterion from healthy lifestyle programs and may help to create a cultural shift in healthy lifestyle promotion and management among children and youth [17, 36]. While our study did not measure stigma, it is reasonable to consider the removal of a weight criterion may have lowered some barriers to enrolment. The influence of stigma on child obesity program enrolment and engagement warrants further investigation.

Our study compared parent-reported child height and weight with facilitator-measured data and the proportion of parents who considered their child’s weight status as a serious health concern. These measures were used as indicators of parental recognition of their child’s weight and the extent to which parents considered overweight as a serious health issue. Our results show where there was disagreement between parent-reported and facilitator-measured child height and weight and the latter identified these children as predominantly above their healthy weight. It is important to recognise that parent-reported data may not have been deliberately inaccurate; the discrepancy in time between enrolment and attendance (i.e., where facilitator measures were taken) may have been enough to push children from the upper limit of one weight category into the next. However, a more likely explanation of this disagreement is that some parents may not have been be aware of their child’s true weight status, as is often reported in overweight and obesity literature [37]. Reasons for this misreporting that have been identified in the literature include parent recall error, rounding or guessing (such as underreporting weight and/ or over reporting height), or reporting based on social desirability or social comparisons [38–40].

Children whose parent-reported height and weight were in disagreement with facilitator-measured data were twice as likely to be enrolled into the program with UEC. Taken together, parent-reported data not in agreement with facilitator-measured data and the lower concern for child weight as a health issue, meant that removing the weight criterion allowed for more families with children who were overweight were able to enrol, even if they were less concerned about their child’s weight status. Our results suggest that the existence of a weight criterion may be a barrier to enrolment by parents whose children are overweight, rather than obese, especially so for those parents who may not be aware of their child’s weight status or do not attribute concern to their child’s weight status.

Parents have reported making judgements about their child’s weight status based on social comparisons to other children rather than an absolute scale in multiple studies [14, 41–45]. Such social comparisons can be described, in part, by visual normalisation theory. Visual normalisation theory is a phenomenon that is becoming more widely recognised, whereby it has become increasingly difficult to identify excess weight in ourselves and others because the general population around us has collectively increased in size, and as such we are less likely to undertake compensatory behaviour or action [41, 43–45]. In Queensland where our project was delivered, 64% of adults were above a healthy weight [11], and so the visual normalisation of overweight is likely to be quite high. A 2014 meta-analysis found that child weight is less likely to be underestimated by parents as child BMI increases, so children that are overweight are more likely to be misclassified than children that are obese (and more so compared to morbidly obese) [46].

Parents of overweight children who enrolled into the groups with UEC were less concerned with the seriousness of their child’s weight status, than parents of overweight children in the groups with TEC. This indicates that parents with overweight children are interested in strategies for the health of their child relating to nutrition and being active, however, that concern may not extend explicitly to concern about their child’s weight. A study in Australia has shown that the vast majority of parents with overweight children are not worried about their child’s weight [21]. Weight status is only one indicator of health behaviours which place individuals at risk of chronic disease. National data on diet and physical activity in Australia [47, 48] indicate these health-related behaviours should be addressed in all children, not just those above their healthy weight, because most children irrespective of weight status do not meet national guidelines for these health-related behaviours. Moreover, for children in particular, the benefits of healthy eating and physical activity extend well beyond chronic disease risk and it is likely that more immediate benefits are greater motivators for children and families [25].

Lower parent concern between TEC and UEC phases indicate that the removal of a weight category criterion and changes to program marketing may have made the program more salient to parents who are not concerned with the seriousness of overweight as a health issue for their child. A focus on healthy lifestyle messaging may speak to parental concern for future weight status, and as such could be a better marketing strategy for program implementers than a focus on weight loss messages [45, 49, 50]. Previous qualitative work with PEACH™ QLD parents showed that their child’s health and happiness were viewed as more important than their weight [14], consistent with other studies [51]. Kelleher et al. undertook a systematic review on barriers and enablers for initial and continued attendance at weight management programs [20]. Parents were found to be more concerned with their child’s wellbeing and attaining a healthy lifestyle, rather than focusing on weight loss, as such, programs that focus on improving health behaviours could be more salient for these parents.

PEACH™ program messages were appropriate for children who were within healthy weight range. A proportion of children whose overweight status was classified as under-reported in this study may have never been able to enrol in and attend PEACH™ QLD if UEC was not applied. It is this group which may arguably have the most to gain from such programs as the likelihood of returning to the healthy weight range before co-morbidities have developed is greater for children who are overweight compared to children who are obese [8, 9]. Improved food parenting skills have been shown to have a positive impact on both shaping and maintaining a child’s weight status as well as the quality of their nutritional intake throughout life [52]. Paediatric weight management and prevention programs which focus on establishing healthy behaviours in primary school-aged children through parenting strategies and consistent health messages have the potential to establish lifelong healthy behaviours early. A program that focuses on healthy lifestyle for all children irrespective of weight may reduce the perception of stigma for families. The dual role of primary and secondary prevention of obesity, in a program focused on normalising healthy lifestyle behaviours through parenting, nutrition, and physical activity is likely to impact on chronic disease risk even in the absence of obesity [53, 54]. Parent peer support and knowledge sharing during group sessions is a beneficial part of group-based child obesity programs [20]. However it is not known whether there may be perceived or real differences in needs in group sessions and content for programs which include all children to promote healthy lifestyle behaviours, compared to those which target only children above a healthy weight. However, the median session attendance was 7 sessions during both the TEC and UEC phases, indicating that parents of children above a healthy weight did not attend less during the UEC when healthy weight children were enrolled.

Healthy lifestyle programs are typically funded as part of a campaign to reduce obesity, PEACH™ QLD was no exception to this [55]. It is important, then, to consider the implications of the absence of weight based enrolment criteria on service planning and provision. Currently in Australia, there is no mechanism through the primary health care system to provide paediatric weight management services publicly in absence of a co-morbidity. In the secondary and tertiary health care system, children are prioritised among busy practitioner caseloads based on acute need [56]. The program therefore, may require a dual communication strategy. One to its target participants, which focuses on healthy lifestyle, and one to funders, which rationalises this approach through the monitoring, reporting and communication of a range of resultant health outcomes including weight. This further reinforces the collection of anthropometric data as a routine health measure rather than of object of shame and embarrassment.

Our cross-sectional study was possible through a natural experiment, and as such our results should be interpreted with caution as this is a secondary analysis of evaluation data which were not originally intended for this purpose. Under the existing evaluation framework data were not collected for a range of factors that could also affect the likelihood of overweight children enrolling to groups with UEC. For example, we are unable to explore the reasons for those who enquired about PEACH™ QLD and were eligible, but chose not to enrol as these were not captured. We cannot say if the dynamics of the group parent sessions were altered because of having parents with children within their healthy weight range participating, however, the higher number of sessions attended during UEC seems to indicate that it did not act as a deterrent to those who ever attended. Conversely, there was a higher number of children that never attended any sessions under UEC, indicating the complexity of engagement in such programs. Differences between child weight status calculated from parent-reported and facilitator-measured height and weight measures may be a result of many factors. For example, the time difference between enrolling (parent-reported measurements) and attending the program (facilitator-measured measurements) may have resulted in a ‘long time lag’ [57], or parent estimation or use of non-standardized instruments. Hence the agreement between child weight statuses at these time points may be influenced by natural variation including regression towards the mean [58]. Consequently we cannot measure if or how much parental bias influenced child weight status calculated from parent-reported data.

The significance level of comparing parental Indigeneity status between eligibility criteria phases should be interpreted with caution due to the large difference in the proportion of not stated and/ or not recorded status between stages. However, it warrants further study if healthy lifestyle messaging is more salient than weight management for Aboriginal and/or Torres Strait Islander parents.

The results of parent concern about child’s weight should be interpreted with caution, as many parents would not consider a child who is overweight (but not obese) as a ‘serious health condition’ however it is not known whether they were aware that their child is overweight or may have perceived them as being a healthy weight. The timeframe to test the UEC (within 1 year) was much shorter than the TEC timeframe (more than 2 years), and results may have been different if the UEC phase was longer. A major limitation is the absence of a control group. The UEC phase began in the third year of the project and therefore the results may be attributable to the maturity of the program and greater awareness of this among the community and health professionals referring families to the program.

## Conclusions

The prevention and treatment of childhood obesity is a wicked problem requiring multiple complementary strategies and political will to deliver effective and sustainable interventions well into the future. The physical and food environments we live in influence the prevalence of childhood obesity and healthy lifestyle programs have the potential to support families to navigate the current obesogenic environment in Australia, and limit its impact on their health. Our findings suggest that families with overweight children are more likely to enrol in a healthy lifestyle program without a weight criterion, where marketing is aimed at improving healthy lifestyle behaviours, than a weight management program with targeted eligibility criteria and recruitment materials focusing on healthy weight. Consideration of removing a weight criterion for group-based programs, as a way of reducing barriers to program enrolment for families with overweight children in particular, warrants further investigation in the collective public health efforts to minimise childhood obesity and establish lifelong healthy habits. These findings suggest a universal weight criterion may be appropriate for scaling up programs into health services, and may in fact save time and resources associated with weight screening. This is an important consideration for policy makers and service providers in ensuring services reach and engage families with the greatest need and opportunity to benefit.

## Additional file

Additional file 1: **Table S1.** Cross-tabulation of parental perception of the seriousness of their child’s weight by eligibility criteria at enrolment, using only data from children with facilitator-measured data. Parental perception of the seriousness of their child’s weight by selection criteria at enrolment, children with measured weight only. **Table S2.** Cross-tabulation of parental perception of the seriousness of their child’s weight by eligibility criteria at enrolment, using parent-reported data to calculate weight category. Parental perception of the seriousness of their child’s weight by selection criteria at enrolment, using parent-reported weight. (DOCX 27 kb)

## Abbreviations

ARIA: Accessibility/ Remoteness Index of Australia
BMI: Body mass index
IOTF: International Obesity Task Force
PEACH™: Parenting, Eating, and Activity for Child Health
QLD: Queensland
SEIFA: Socioeconomic Indexes for Areas
SES: Socioeconomic status
TEC: Targeted Eligibility Criterion, all Queensland children aged 5–11 years were eligible to enrol, so long as they were above their healthy weight
UEC: Universal Eligibility Criterion, all Queensland primary school children were eligible to enrol, regardless of their weight status

## References

[1] Australian Health Survey: updated results, 2011–2012. Australian Bureau of Statistics (ABS). Canberra; 2013. http://www.abs.gov.au/ausstats/abs@.nsf/Lookup/4364.0.55.003main+features12011-2012. Accessed 20 Jan 2018

[2] Halfon Neal, Larson Kandyce, Slusser Wendy (2013). Associations Between Obesity and Comorbid Mental Health, Developmental, and Physical Health Conditions in a Nationally Representative Sample of US Children Aged 10 to 17. Academic Pediatrics.

[3] Neovius M., Sundstrom J., Rasmussen F. (2009). Combined effects of overweight and smoking in late adolescence on subsequent mortality: nationwide cohort study. BMJ.

[4] Sanders Ross H., Han Ahreum, Baker Julien S., Cobley Stephen (2015). Childhood obesity and its physical and psychological co-morbidities: a systematic review of Australian children and adolescents. European Journal of Pediatrics.

[5] World Health Organisation (2016). Consideration of the evidence on childhood obesity for the commission on ending childhood obesity.

[6] Stokes Andrew, Ni Yu, Preston Samuel H. (2017). Prevalence and Trends in Lifetime Obesity in the U.S., 1988–2014. American Journal of Preventive Medicine.

[7] Moores Carly J., Miller Jacqueline, Daniels Lynne A., Vidgen Helen A., Magarey Anthea M. (2018). Pre–post evaluation of a weight management service for families with overweight and obese children, translated from the efficacious lifestyle intervention Parenting, Eating and Activity for Child Health (PEACH). British Journal of Nutrition.

[8] Ochner Christopher N., Barrios Dulce M., Lee Clement D., Pi-Sunyer F. Xavier (2013). Biological mechanisms that promote weight regain following weight loss in obese humans. Physiology & Behavior.

[9] Ochner Christopher N, Tsai Adam G, Kushner Robert F, Wadden Thomas A (2015). Treating obesity seriously: when recommendations for lifestyle change confront biological adaptations. The Lancet Diabetes & Endocrinology.

[10] Magarey A. M., Perry R. A., Baur L. A., Steinbeck K. S., Sawyer M., Hills A. P., Wilson G., Lee A., Daniels L. A. (2011). A Parent-Led Family-Focused Treatment Program for Overweight Children Aged 5 to 9 Years: The PEACH RCT. PEDIATRICS.

[11] Queensland Health (2016). The Health of Queenslanders 2016. Report of the Chief Health Officer Queensland.

[12] Welsby D, et al. Process evaluation of an up-scaled community based child obesity treatment program: NSW Go4Fun®. BMC Public Health. 2014. 10.1186/1471-2458-14-140. 10.1186/1471-2458-14-140 PMC3923092 24512080

[13] Lobstein Tim, Jackson-Leach Rachel (2006). Estimated burden of paediatric obesity and co-morbidities in Europe. Part 2. Numbers of children with indicators of obesity-related disease. International Journal of Pediatric Obesity.

[14] Davidson K, Vidgen H. Why do parents enrol in a childhood obesity management program?: a qualitative study with parents of overweight and obese children. BMC Public Health. 2017. 10.1186/s12889-017-4085-2. 10.1186/s12889-017-4085-2 PMC5290615 28153053

[15] Croyden DL, et al. A narrative account of implementation lessons learnt from the dissemination of an up-scaled state-wide child obesity management program in Australia: PEACH™ (parenting, eating and activity for child Health) Queensland. BMC Public Health. 2018. 10.1186/s12889-018-5237-8. 10.1186/s12889-018-5237-8 PMC5851159 29534700

[16] Lydecker Janet A., O'Brien Elizabeth, Grilo Carlos (2017). Parents’ Implicit and Explicit Attitudes Towards Childhood Obesity. Journal of Adolescent Health.

[17] Puhl Rebecca M., Heuer Chelsea A. (2010). Obesity Stigma: Important Considerations for Public Health. American Journal of Public Health.

[18] Zenlea I. S., Thompson B., Fierheller D., Green J., Ulloa C., Wills A., Mansfield E. (2017). Walking in the shoes of caregivers of children with obesity: supporting caregivers in paediatric weight management. Clinical Obesity.

[19] Abbott Rebecca A, Lee Amanda J, Stubbs Christina O, Davies Peter SW (2010). Accuracy of weight status perception in contemporary Australian children and adolescents. Journal of Paediatrics and Child Health.

[20] Kelleher E., Davoren M. P., Harrington J. M., Shiely F., Perry I. J., McHugh S. M. (2016). Barriers and facilitators to initial and continued attendance at community-based lifestyle programmes among families of overweight and obese children: a systematic review. Obesity Reviews.

[21] Lampard Amy M., Byrne Susan M., Zubrick Stephen R., Davis Elizabeth A. (2008). Parents’ concern about their children's weight. International Journal of Pediatric Obesity.

[22] Moores CJ, et al. CONSORT to community: translation of an RCT to a large-scale community intervention and learnings from evaluation of the upscaled program. BMC Public Health. 2017. 10.1186/s12889-017-4907-2. 10.1186/s12889-017-4907-2 PMC5708099 29187157

[23] GOLLEY Rebecca K., PERRY Rebecca A., MAGAREY Anthea, DANIELS Lynne (2007). Family-focused weight management program for five- to nine-year-olds incorporating parenting skills training with healthy lifestyle information to support behaviour modification. Nutrition & Dietetics.

[24] National Health & Medical Research Council (NHMRC) (2013). Clinical practice guidelines for the management of overweight and obesity in adults, adolescents and children in Australia.

[25] NHMRC (2013). Australian Dietary Guidelines Summary.

[26] Commonwealth of Australia (2014). Australia’s physical activity and sedentary behaviour guidelines, 5–12 years.

[27] Australian Institute of Health and Welfare (AIHW) (2010). National best practice guidelines for collecting indigenous status in health data sets.

[28] Australian Bureau of Statistics (ABS) (2013). Socio-economic indexes for areas (SEIFA) 2011.

[29] ABS (2013). Accessibility/remoteness index of Australia (ARIA2011+).

[30] A SAS Program for the 2000 CDC growth charts (ages 0 to 20 years), Table 1. Centers for Disease Control and Prevention, Atlanta. 2016. https://www.cdc.gov/nccdphp/dnpao/growthcharts/resources/sas.htm. Accessed 1 June 2017.

[31] Cole T. J., Lobstein T. (2012). Extended international (IOTF) body mass index cut-offs for thinness, overweight and obesity. Pediatric Obesity.

[32] Dahly Darren L., Gordon-Larsen Penny, Popkin Barry M., Kaufman Jay S., Adair Linda S. (2009). Associations between Multiple Indicators of Socioeconomic Status and Obesity in Young Adult Filipinos Vary by Gender, Urbanicity, and Indicator Used. The Journal of Nutrition.

[33] Hubbard Alan E., Ahern Jennifer, Fleischer Nancy L., Laan Mark Van der, Lippman Sheri A., Jewell Nicholas, Bruckner Tim, Satariano William A. (2010). To GEE or Not to GEE. Epidemiology.

[34] Senterre C, Levêque A, Vanthournout B, Dramaix M. Does the presence of siblings affect the results produced by a surveillance system of child mistreatment? Comparisons of several commonly-used statistical methods. BMC Res Notes. 2015. 10.1186/s13104-015-1710-y. 10.1186/s13104-015-1710-y PMC4673765 26646906

[35] Pont Stephen J., Puhl Rebecca, Cook Stephen R., Slusser Wendelin (2017). Stigma Experienced by Children and Adolescents With Obesity. Pediatrics.

[36] Tomiyama AJ, et al. How and why weight stigma drives the obesity ‘epidemic’ and harms health. BMC Med. 2018. 10.1186/s12916-018-1116-5. 10.1186/s12916-018-1116-5 PMC6092785 30107800

[37] Merema Matt R., Sullivan Denise L., Pollard Christina M., Abraham Jillian A., Tomlin Stephania M., Radomiljac Ali L. (2015). Parents' perception of their child's weight status and intention to intervene: a Western Australian cross-sectional population survey, 2009-12. Australian and New Zealand Journal of Public Health.

[38] Weden Margaret M., Brownell Peter B., Rendall Michael S., Lau Christopher, Fernandes Meenakshi, Nazarov Zafar (2013). Parent-Reported Height and Weight as Sources of Bias in Survey Estimates of Childhood Obesity. American Journal of Epidemiology.

[39] Brettschneider Anna-Kristin, Ellert Ute, Schaffrath Rosario Angelika (2012). Comparison of BMI Derived from Parent-Reported Height and Weight with Measured Values: Results from the German KiGGS Study. International Journal of Environmental Research and Public Health.

[40] Akinbami Lara J., Ogden Cynthia L. (2009). Childhood Overweight Prevalence in the United States: The Impact of Parent-reported Height and Weight. Obesity.

[41] Robinson E., Kersbergen I. (2017). Overweight or about right? A norm comparison explanation of perceived weight status. Obesity Science & Practice.

[42] Cornelissen Katri K., Gledhill Lucinda J., Cornelissen Piers L., Tovée Martin J. (2016). Visual biases in judging body weight. British Journal of Health Psychology.

[43] Robinson E. (2017). Overweight but unseen: a review of the underestimation of weight status and a visual normalization theory. Obesity Reviews.

[44] Robinson E., Sutin A. R. (2016). Parental Perception of Weight Status and Weight Gain Across Childhood. PEDIATRICS.

[45] Hansen A. R., Duncan D. T., Tarasenko Y. N., Yan F., Zhang J. (2014). Generational Shift in Parental Perceptions of Overweight Among School-Aged Children. PEDIATRICS.

[46] Lundahl A., Kidwell K. M., Nelson T. D. (2014). Parental Underestimates of Child Weight: A Meta-analysis. PEDIATRICS.

[47] HARDY LOUISE L., BARNETT LISA, ESPINEL PAOLA, OKELY ANTHONY D. (2013). Thirteen-Year Trends in Child and Adolescent Fundamental Movement Skills. Medicine & Science in Sports & Exercise.

[48] Mihrshahi Seema, Myton Rimma, Partridge Stephanie R., Esdaile Emma, Hardy Louise L., Gale Joanne (2018). Sustained low consumption of fruit and vegetables in Australian children: Findings from the Australian National Health Surveys. Health Promotion Journal of Australia.

[49] Clark H. R., Goyder E., Bissell P., Blank L., Peters J. (2007). How do parents' child-feeding behaviours influence child weight? Implications for childhood obesity policy. Journal of Public Health.

[50] Parkinson K N, Drewett R F, Jones A R, Dale A, Pearce M S, Wright C M, Adamson A J (2011). When do mothers think their child is overweight?. International Journal of Obesity.

[51] Syrad H., Falconer C., Cooke L., Saxena S., Kessel A. S., Viner R., Kinra S., Wardle J., Croker H. (2014). ‘Health and happiness is more important than weight’: a qualitative investigation of the views of parents receiving written feedback on their child's weight as part of the National Child Measurement Programme. Journal of Human Nutrition and Dietetics.

[52] Gerards S. M. P. L., Kremers S. P. J. (2015). The Role of Food Parenting Skills and the Home Food Environment in Children’s Weight Gain and Obesity. Current Obesity Reports.

[53] Nyberg G, Norman Å, Sundblom E, Zeebari Z, Schäfer Elinder L. Effectiveness of a universal parental support programme to promote health behaviours and prevent overweight and obesity in 6-year-old children in disadvantaged areas, the healthy school start study II, a cluster-randomised controlled trial. Int J Behav Nutr Phys Act. 2016. 10.1186/s12966-016-0327-4. 10.1186/s12966-016-0327-4 PMC4721005 26795378

[54] Olsen NJ, et al. The healthy start project: a randomized, controlled intervention to prevent overweight among normal weight, preschool children at high risk of future overweight. BMC Public Health. 2012. 10.1186/1471-2458-12-590. 10.1186/1471-2458-12-590 PMC3490801 22852799

[55] Council of Australian Governments (COAG) (2018). National Partnership Agreement on preventive Health.

[56] Paediatric Obesity Working Group (2017). An integrated approach for tackling childhood overweight and obesity in Queensland.

[57] Gorber S. Connor, Tremblay M., Moher D., Gorber B. (2007). A comparison of direct vs. self-report measures for assessing height, weight and body mass index: a systematic review. Obesity Reviews.

[58] Cockrell Skinner Asheley, Goldsby TaShauna U., Allison David B. (2016). Regression to the Mean: A Commonly Overlooked and Misunderstood Factor Leading to Unjustified Conclusions in Pediatric Obesity Research. Childhood Obesity.

