# Supplementary material for: Enrolment of families with overweight children into a program aimed at reducing childhood obesity with and without a weight criterion: a natural experiment
Source: BMC Public Health. 2019 Jun 14;19:756. doi: 10.1186/s12889-019-6894-y (PMC6570944; doi:10.1186/s12889-019-6894-y)
Supplement: Supplementary file 1 — Table S1. Cross-tabulation of parental perception of the seriousness of their child’s weight by eligibility criteria at enrolment, using only data from children with facilitator-measured data. Parental perception of the seriousness of their child’s weight by selection criteria at enrolment, children with measured weight only. Table S2. Cross-tabulation of parental perception of the seriousness of their child’s weight by eligibility criteria at enrolment, using parent-reported data to calculate weight category. Parental perception of the seriousness of their child’s weight by selection criteria at enrolment, using parent-reported weight. (DOCX 27 kb) [file 12889_2019_6894_MOESM1_ESM.docx]

**Additional file 1**

**Table S1**: Cross-tabulation of parental perception of the seriousness of their child’s weight by eligibility criteria at enrolment, using only data from children with facilitator-measured data

Parental perception of the seriousness of their child’s weight by selection criteria at enrolment, children with measured weight only

|  | | **Do you think that your child's weight is a serious health condition?** | | | | | | | | |
| --- | --- | --- | --- | --- | --- | --- | --- | --- | --- | --- |
|  |  | **All**  **(*n* = 662)** | | | **Overweight children**  **(*n* = 138)** | | | **Obese children**  **(*n* = 524)** | | |
|  | | Not serious | Serious | Very serious | Not serious | Serious | Very serious | Not serious | Serious | Very serious |
| **Targeted eligibility criteria** |  |  |  |  |  |  |  |  |  |  |
|  | *n* (%)^‡^ | 125 (25.2) | 184 (37.0) | 188 (37.8) | 40 (44.4) | 36 (40.0) | 14 (15.6) | 85 (20.9) | 148 (36.4) | 174 (42.8) |
|  | AR^1^ | -2.0 | 1.6 | 0.5 | -2.4 | 2.5 | 0.5 | 0.1 | 0.4 | -0.5 |
| **Universal eligibility criteria** |  |  |  |  |  |  |  |  |  |  |
|  | *n* (%)^‡^ | 56 (33.9) | 50 (30.3) | 59 (35.8) | 32 (66.7) | 10 (20.8) | 6  (12.5) | 24 (20.8) | 40 (34.2) | 53 (45.3) |
|  | AR^1^ | 2.0 | -1.6 | -0.5 | 2.4 | -2.5 | -0.5 | - 0.1 | -0.4 | 0.5 |
|  | | **χ2 = 5.224, df = 2*, p* = 0.081** | | | **χ2 = 6.615, df = 2, *p* = 0.044** | | | **χ2 = 0.262, df = 2, *p* = 0.885** | | |

*p* values obtained using second-order Rao-Scott adjusted chi-square statistic.

‡ Row percentages

*^1^AR adjusted residuals, ARs over 2 and below −2 indicate statistically significant differences.*

**Table S2**: Cross-tabulation of parental perception of the seriousness of their child’s weight by eligibility criteria at enrolment, using parent-reported data to calculate weight category

Parental perception of the seriousness of their child’s weight by selection criteria at enrolment, using parent-reported weight

|  | | **Do you think that your child's weight is a serious health condition?** | | | | | | | | |
| --- | --- | --- | --- | --- | --- | --- | --- | --- | --- | --- |
|  |  | **All**  **(*n* = 710)** | | | **Overweight children**  **(*n* = 166)** | | | **Obese children**  **(*n* = 544)** | | |
|  | | Not serious | Serious | Very serious | Not serious | Serious | Very serious | Not serious | Serious | Very serious |
| **Targeted eligibility criteria** |  |  |  |  |  |  |  |  |  |  |
|  | *n* (%)^‡^ | 145 (26.8) | 202 (37.3) | 194 (35.9) | 55 (45.5) | 46 (38.0) | 20 (16.5) | 90 (21.4) | 156 (37.1) | 174 (41.4) |
|  | AR^1^ | -1.6 | 1.6 | -0.1 | -1.4 | 0.8 | 0.9 | -0.6 | 1.3 | -0.7 |
| **Universal eligibility criteria** |  |  |  |  |  |  |  |  |  |  |
|  | *n* (%)^‡^ | 56 (33.1) | 52 (30.8) | 61 (36.1) | 26 (57.8) | 14 (31.1) | 5  (11.1) | 30 (24.2) | 38 (30.6) | 56 (45.2) |
|  | AR^1^ | 1.6 | -1.6 | 0.1 | 1.4 | -0.8 | -0.9 | 0.6 | -1.3 | 0.7 |
|  | | **χ2 = 3.380, df = 2*, p* = 0.185** | | | **χ2 = 2.093, df = 2, *p* = 0.350** | | | **χ2 = 1.781, df = 2, *p* = 0.414** | | |

*p* values obtained using second-order Rao-Scott adjusted chi-square statistic.

‡ Row percentages

*^1^AR adjusted residuals, ARs over 2 and below −2 indicate statistically significant differences.*
